# Perceptual influence of elementary three-dimensional geometry: (2) fundamental object parts

**DOI:** 10.3389/fpsyg.2015.01427

**Published:** 2015-09-24

**Authors:** Minija Tamosiunaite, Rahel M. Sutterlütti, Simon C. Stein, Florentin Wörgötter

**Affiliations:** ^1^Faculty of Physics – Biophysics and Bernstein Center for Computational Neuroscience, University of GöttingenGöttingen, Germany; ^2^Department of Informatics, Vytautas Magnus UniversityKaunas, Lithuania

**Keywords:** object parts, visual assessment, 3D-perception, point-clouds, concave-convex

## Abstract

Objects usually consist of parts and the question arises whether there are perceptual features which allow breaking down an object into its *fundamental parts* without any additional (e.g., functional) information. As in the first paper of this sequence, we focus on the division of our world along convex to concave surface transitions. Here we are using machine vision to produce convex segments from 3D-scenes. We assume that a fundamental part is one, which we can easily name while at the same time there is no natural subdivision possible into smaller parts. Hence in this experiment we presented the computer vision generated segments to our participants and asked whether they can identify and name them. Additionally we control against segmentation reliability and we find a clear trend that reliable convex segments have a high degree of name-ability. In addition, we observed that using other image-segmentation methods will not yield nameable entities. This indicates that convex-concave surface transition may indeed form the basis for dividing objects into meaningful entities. It appears that other or further subdivisions do not carry such a strong semantical link to our everyday language as there are no names for them.

## 1. Introduction

Humans have very far-reaching abilities to recognize, design, and manipulate complex objects and those are often composed of several parts. It remains, however, unknown how we break down an object into its parts, especially in view of the fact that the parts which we recognized can be considered as objects by themselves most of the time. E.g., a chair can be composed of legs, seat, backrest, etc. The divisions into parts, which we perform, ends usually at entities, which for us still have some (functional) meaning. Hence, do we not divide a chair-leg again into two (or more) parts, even if—for example—the colors of top and bottom of the leg differ^1^. Thus, it seems that many times we perform part-divisions such that we end up at “fundamental parts” to which we still can attach some semantics. As an adult you could use your knowledge about structure and function of object-parts to do this. But this cannot be true for very young infants, which soon after birth grasp a toy hammer either at the head or the handle, but not at the junction of head and handle. Thus, above the age of about 3 months infants have no problems to individuate and successfully grasp parts, which still have no functional meaning to them (Jeannerod, 1994).

This indicates that there might be fundamental perceptual priors existing on which the concept of “what is a fundamental part” can rely, independently of functional semantics. Commonly one assumes that part-identification (and recognition) requires complex—innate as well as acquired—cognitive processes (Mandler, 1992, 2012; Carey, 2011), leading to multifactorial representations in the neural system (Riesenhuber and Poggio, 2000; Palmeri and Gauthier, 2004). However, it remains unclear how objects can be segregated into parts, and identified given the high degree of variability of the sensory features which arise even from similar objects (Geisler, 2008).

In the first paper of this sequence (Wörgötter et al., 2015) we had focused on the question to what degree convex-concave surface transitions may form the basis for our assessment of object-ness (object-goodness is a synonym for this). This study had been triggered by earlier works that had suggested that convex-concave surface transitions influence how we perceive objects (Rubin, 1958; Koenderink and van Doorn, 1982; Hoffman and Richards, 1984; Biederman, 1987; Braunstein et al., 1989; Cate and Behrmann, 2010; Bertamini and Wagemans, 2013). We had observed that people prefer compact and convex 3D-objects, hence those with few concavities. A detailed discussion of the literature had been provided in the first paper (Wörgötter et al., 2015), too, which shall not be repeated here.

The current study continues investigating these aspects and here we ask to what degree will convex-concave surface transitions lead to a perceptual division of our real 3D-world into “fundamental parts”? Starting point of this investigation is a computer vision algorithm that segments scenes without additional knowledge—hence in a purely data-driven way—into convex entities. The efficiency of this algorithms had been demonstrated in a set of technical papers (Stein et al., 2014a,b; Schoeler et al., 2015) and it can, thus, serve as a basis for creating ground-truth convex segments. Hence computer vision is not in the core of this study, instead we are asking: do these convex segments carry any “meaning” for us? Thus, is there a connection of a purely data driven bottom up (artificially emulated) perceptual process—the breaking up of the world into convex entities—with aspects of conscious cognition? Following the discussion above about chairs and chair-legs and considering the fact that we do not easily continue subdividing a chair-leg into smaller meaningful entities, we assume that for us a fundamental meaningful part is one which we can *naturally* name and which we cannot *naturally* divide any further into smaller parts, which have still have a name. Hence “name-ability” is in this study the measure for an entity which has for us still a meaning and we will show that convex-concave surface transitions subdivide real 3D-scene into (mostly) nameable entities, which will not happen for any other type of subdivision (e.g., subdivision by color, texture, etc.). We are aware of the possibility that there might be other aspects by which “meaning” of a segment could be assigned, for example “graspability of a segment” and in Section 3 we are addressing some of the complex questions that arise from the here-chosen name-ability paradigm. Clearly, this study does not attempt to capture each and every aspect of object-part semantics but tries to show that there is indeed a strong correlation between bottom up-segmented convex entities and our ability to give a name to and, thus, understandİ these segments.

## 2. Experiment - visual scene analysis for part recognition

This experiment asks: Do real-world entities, which are obtained by splitting 3D-scenes along concave/convex transitions correspond to those entities for which we have a name? Hence, which are for us in some sense a fundamental object-part.

### 2.1. Methods

#### 2.1.1. Visual stimuli and pre-processing

A total of 10 real scenes have been analyzed, all of which are shown in **Figure 3**, left panels. Scenes consist of 3D-point cloud data and the corresponding RBG image. In general all scenes were recorded by RGB-D sensors (e.g., “Kinect”), which provide 3D-point cloud data and matched 2D RGB image. They were taken from openly available machine vision data bases (Richtsfeld et al., 2012; Silberman et al., 2012). The spatial resolution of the Kinect sensor falls in the depth range of 0.6 m to about 3.0–5.0 m. This limits the types of scenes that can be used. The here used indoor scenes are a well established and very difficult benchmark set for current machine vision approaches (Richtsfeld et al., 2012; Silberman et al., 2012).

**Figure 1:**
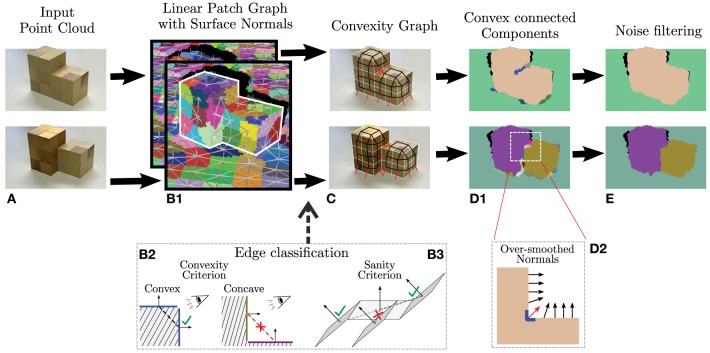
**Overview of the computer vision method use for convex 3D-scene segmentation (for details see Stein et al., 2014a,b)**. **(A)** Two test objects **(B1)** Initial point clouds are reduced to supervoxels (Papon et al., 2013) with graph edges showing how voxels are neighbors. **(B2)** Conventional definition of convex and concave configurations. **(B3)** Singular locations like the one shown are not treated as concave, which massively improves algorithmic performance **(C)** Resulting convex (black) and concave (red) connectivity graph. **(D1)** Segmentation. **(D2)** Noise reduction mechanisms avoiding over-smoothing inside corners. **(E)** Final segmentation. Figure modified from Stein et al. (2014b).

We segmented the scenes along convex-concave transitions in the 3D-data by a machine vision algorithm. Figure 1 provides an overview of this method shown by ways of two simple test objects (Figure 1A). Point clouds are first reduced to few so-called supervoxels (Papon et al., 2013) which capture the scene geometry by their neighborhood relations (graph-edges in Figure 1B1). Convex and concave edge configuration are found using a conventional criterium (Figure 1B2) employed at the surface normals of each point but corrected against singularities as shown in Figure 1B3. (Some surface normals are shown graphically in Figure 1D2 by ways of arrows.) This results is convex (black) and concave (red) connections (Figure 1C), which are used to break up the scene (Figure 1D1). Corners such as the one shown in Figure D2 lead to an over-smoothing of the normals (see red arrow) and the algorithm at the end corrects for this leading to the final segmentation as shown in Figure 1E. Details of the algorithm are described elsewhere (Stein et al., 2014a,b). Note, this is a model-free, purely data-driven segmentation algorithm, as required for the purpose of this study, which does not use any additional features for segmentation. Due to the limited spatial resolution of the RGB-D sensors, small objects cannot be consistently labeled. Thus, segments smaller than 0.3% of the image size were manually blackened out by us as they most often represent sensor noise, and the same was done with reflecting surfaces, which the Kinect sensor cannot measure. After this we received a total of 247 segments (i.e., about 20–30 per image). Segments are labeled on the 2D RGB image with different colors to make them distinguishable for the observer.

#### 2.1.2. Participants and procedures

Participants were 20 healthy adults (age: 22–35) the purpose of this study had not been revealed to them but all experimental procedures had been clearly explained. Participants only partook in the experiment after having given their explicit consent. The experiment is not harmful and no sensitive data had been recorded and experimental data has been treated anonymously and only the instructions explained below had been given to the participants. The experiment was performed in accordance with the ethical standards laid down by the 1964 Declaration of Helsinki. We followed the relevant guidelines of the Germany Psychological Society according to which this experiment, given the conditions explained above, does not need explicit approval by an Ethics Committee (Document: 28.09.2004 DPG: “Revision der auf die Forschung bezogenen ethischen Richtlinien.”

For the experiment we asked our subjects to compare the segmented, color-labeled scenes with the corresponding original RGB image (total amount of data: 4940). Segments were one by one highlighted in the labeled image and, for every segment, we asked our subjects to look at the original RGB image, find the corresponding region asking: *“How precisely can you name it?”*; and recorded their utterances for later analysis.

Note, the reverse procedure of asking subjects to label the objects seen in the RGB images and then comparing it to the algorithmic analysis is fundamentally flawed in the context of this study as in this case subjects will use their world-knowledge and label objects according to their most prevalent (the most “natural”) higher level concepts. E.g., when looking at the image of a woman, instead of labeling body parts, subjects will generally label the complete figure as “woman” (see Silberman et al., 2012, for such an approach).

#### 2.1.3. Data analysis and statistical tests

Subsequently we analyzed the utterances and divided them into three groups: (1) precise naming of a segment (e.g., “table leg”), where it does not play a role whether or not subjects would use unique names (e.g., “table leg,” “leg,” and “table support” are equally valid). (2) definite failure/impossibility to name a segment. (3) potentially non-fundamental segments, where subjects stated that they think this is segment could still be further divided or that this an object but that he/she is not sure about the identification (about its name); e.g., for too small segments.

In general we recorded and analyzed the complete utterances that participants made. Case 1 and 2 led always to brisk statements (either a name was quickly given to the segment, or participants clearly said that this segment cannot be named). Case 3 covered essentially the remainder of the utterances where participants began to engage in more or less lengthy interpretative discourse about the viewed segment. When this happened we always counted this as a case 3.

For quantitative analysis we are, in addition, controlling for errors introduced by image acquisition and/or by the computer vision algorithm. For this we use the known distance error function of the Kinect sensor (Smisek et al., 2011) to calculate the reliability of every segment as described next:

Let *x* be a segment consisting of *N*_*x*_ point-cloud points at distances *z*. Reliability *R*_*x*_ is calculated as $R_{x}=\frac{100\;q(1)}{A_{x}[q(z)]}$ where $A_{x}[q(z)]=\frac{1}{N_{x}}\sum_{i=0}^{N_{x}}q(z_{i})\;$ is the average discretization error and *q*(*z*) = 2.73*z*^2^ + 0.74*z* − 0.58 the known error function (Smisek et al., 2011). This measure is normalized to 1m distance and yields 100 for a planar vertical segment at this distance, smaller values for larger distances and vice versa.

The intuition behind this error function is simple: Given the known Kinect error function (Smisek et al., 2011) we created here a function that counts how far away a segment is (the farther the worse) and how big it is (the bigger the better) and balanced these two terms against each other to provide the so-called segment's reliability. This creates a reliability weighing that is similar to our own visual experience, where we find it easier to recognize large-nearby objects than those that are far away and small.

After this procedure we plotted the counts in groups 1, 2, and 3 against the reliability value of the respective segments as scatter plots were we show all raw data as well as mean values and regression lines across reliability intervals [0, 10];[10, 20];···;[150, 160] plotted above their interval centers. Note, we resorted to plot raw data as scatter plots instead of mean+standard deviation because this shows better the data density for both axes.

It is important to comment here on the issue of potential controls for this study. Those could theoretically be obtained by using other feature-based (low-level, data-driven) image segmentation methods, for example using the same images segmented by a state-of-the-art color segmentation method or any other low-level, data-driven segmentation. It is a known, difficult problem in computer vision that none of these methods will produce anything “object-like.” For example, color-based segmentation yields highly luminance dependent results, as is clearly visible from visual inspection of the middle panels in **Figure 3**. In the discussion section we will discuss this aspect in detail, which makes it impossible to use any other data-driven method for comparison. Trying to name segments obtained by such methods just leads to nothing. Higher level, model-based segmentation approaches, which use human-labeled data, will indeed lead to nameable segments (Silberman et al., 2012), but these methods are not anymore data-driven and can therefore also not be compared to our approach.

### 2.2. Results

We employed a “dumb,” model-free computer vision algorithm that splits 3D-scenes along concave-convex transitions (Stein et al., 2014a,b) asking to what degree does this low-level segmentation yield identifiable entities? Note, we are not concerned with object recognition or categorization here, instead we wanted to know whether this fundamental geometric segmentation leads to entities, which can be individuated and understood by us as meaningful parts.

**Figure 2:**
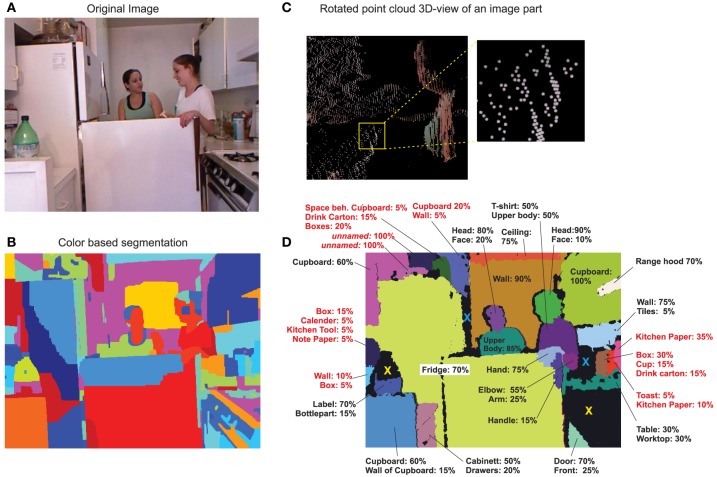
**Humans can with high reliability identify image segments that result from splitting images along concave-convex surface transitions**. **(A)** One example scene used for analysis. **(B)** Color-based segmentation of the scene. **(C)** Point cloud image of parts of the scene (rotated 3D view) with RGB data overlayed. **(D)** 3D-segmented scene and segment names used by our subjects to identify objects. Missing percentages are the non-named cases. E.g., the pink segment top-left was named “cupboard” by 60% of the subjects and remained unidentified by the remaining 40%. Red lettering indicates segments with reliability less than 50.

**Figure 3:**
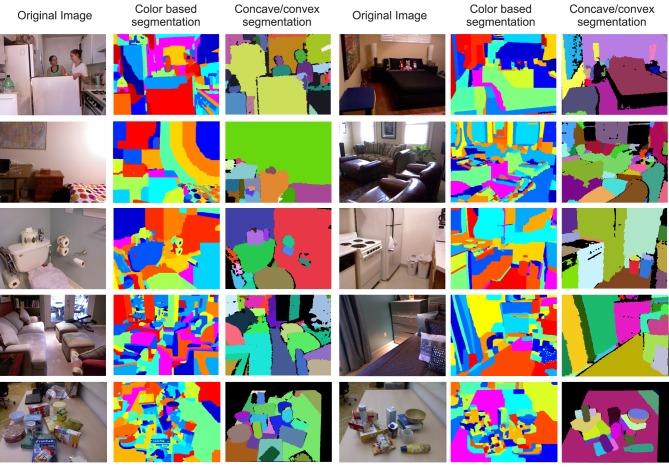
**Left panels show all visual scenes (RGB images only) used for Experiment 2 of this study and their segmentations**. Scenes have been segmented by a state-of-the-art, bottom-up segmentation algorithm which uses color similarities (Ben Salah et al., 2011) and the results show that these segments rarely correspond to objects (middle panels). Note, it is possible to train classifiers with object models or partial models to obtain segmentation of complex, compound objects also in such scenes (Richtsfeld et al., 2012; Silberman et al., 2012; Ückermann et al., 2012). This, however, requires a human-defined training set. Different from this, here we are strictly concerned with model-free, bottom-up object segmentation. The here used 3D-segmentation, back-projected onto the images, is shown in the right panels.

One example scene is shown in Figure 2A recorded with an RGB-D sensor (“Kinect”), which produces 3D-point cloud data. All other scenes are of equal complexity (Figure 3). Using an advanced, model-free *color-based* segmentation method (Ben Salah et al., 2011) one can see that the resulting image segments rarely correspond to objects in the scene (Figure 2B) and this is also extremely dependent on illumination (see Figure 3, middle). Unwanted merging or splitting of objects will, *regardless* of the chosen segmentation parameters, generically happen (e.g., “throat+face,” “fridge-fragments,” etc. Figure 2B).

Instead, here 3D-point clouds were segmented along concave/convex transitions. As cloud data is extremely difficult to view and assess (see Figure 2C, for a magnified view), the resulting 3D-segments were back-projected onto the 2D RGB image and color labeled (Figure 2D). Too small segments had been combined and blackened out, some cases are marked by a blue “x” in Figure 2D (same for reflecting surfaces, see e.g., yellow “x,” the stove is indeed too reflecting for the Kinect and, in addition, the few now-reflecting parts which exist at the stove produced here too small segments).

**Figure 4:**
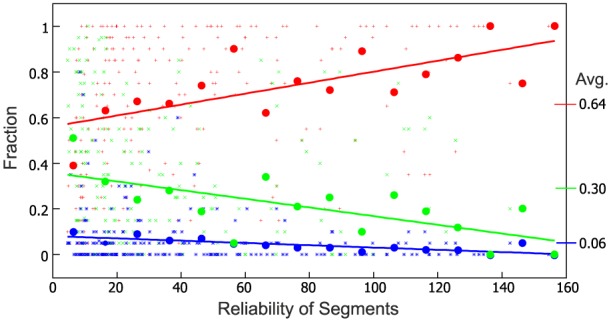
**Fraction of identified (red), not-identified (green) and unclear (blue) segments for the complete data set (20 subjects, 247 segments each) plotted against their reliability**. Fat dots represent averages across reliability intervals [0, 10];[10, 20];···;[150, 160] plotted above their interval centers, lines are the corresponding regression lines. The ability to identify a segment increases with reliability. Grand averages (red: 0.64, green: 0.30, blue: 0.06) for all data are shown, too.

Subjects many times used different names (e.g., “face” or “head”) to identify a segment, which are equally valid as both describe a valid conceptional entity (a part). Several segments could not always be identified, however. Averaging across all data shows that 64% of the segments could be identified, 30% not, and there were 6% potentially cases for further subdivision. Are these 30% counter-examples against our conjecture or are due to machine vision errors? Thus, we additionally considered the *reliability* of the individual segments (see Section 2.1). The Kinect sensor produces a discretization error (Smisek et al., 2011) as can be seen by the stripy patterns in Figure 2C (see also yellow box). Due to this, data at larger distances become quadratically more unreliable (see Section 2.1). As a result, for example, two objects will be combined into one segment just due to the fact that the separating concavity cannot be resolved anymore. When considering reliability we find that subjects could more often identify *reliable* segments (Figure 4, red) and unrecognized cases dropped accordingly (green). Comparing this result again to the segmented example scene (Figure 2D) we find that, indeed, for less reliable segments (red lettering) identification is low or ambivalent as compared to reliable ones.

## 3. Discussion

The hypothesis that concave-convex surface transitions are instrumental for our object understanding is an old one and there are several individual lines of evidence from perception that are supporting this (Rubin, 1958; Koenderink and van Doorn, 1982; Hoffman and Richards, 1984; Biederman, 1987; Braunstein et al., 1989; Cate and Behrmann, 2010; Bertamini and Wagemans, 2013). The experiments reported in the first paper (Wörgötter et al., 2015) tried to address the problem of human object concepts in an abstract way using abstract 3D-geometrical structures (polycubes), hence, independent from the real world. Here we used real scenes and found that convex-concave surface transitions can be used to individuate and name object-parts.

It is of interest to discuss this aspect first from a more technical perspective namely that of computer vision. This field is terrifically hard pressed to segment scenes into object-like entities. It has been possible since years to perform color-, edge-, texture-, etc. based segmentation with increasing success (Comaniciu and Meer, 2002; Felzenszwalb and Huttenlocher, 2004; Boykov and Funka-Lea, 2006; Arbelaez et al., 2011; Ben Salah et al., 2011) but it is known, and discussed in the above cited works, that none of these methods renders anything object- or part-like (see also Figure 2B). Purely data-driven, bottom-up image segmentation seems doomed with respect to this aspect. Computer vision has resorted much to the use of model-based (top-down) approaches, which require the often very tedious learning of large sets of object models (Arbelaez et al., 2012; Richtsfeld et al., 2012; Silberman et al., 2012; Gupta et al., 2013), and the choice of models by the designer will bias the system. Thus, also in this field it is an intriguing problem of how to arrive at a *meaningful* image partitioning.

Several computer vision approaches have also used concave/convex surface transitions for image segmentation (Vaina and Zlateva, 1990; Siddiqi and Kimia, 1995; Rosin, 2000; Moosmann et al., 2009; Richtsfeld et al., 2012; Ückermann et al., 2012), where these algorithms most often had been complemented by additional features to improve segmentation. Recently we were able to design a segmentation algorithm based on this principle, which contained a few important geometrical corrections, and—this way—became strong enough to compete with far more complex segmentation methods for object recognition (Stein et al., 2014a,b; Schoeler et al., 2015). This made it possible to segment scenes in a bottom-up way with few intrinsic/systematic errors and only by this we could begin to ask whether such a partitioning would indeed lead to entities that carry “meaning” for us.

Name-ability, hence the identification of a segment as a unique entity with a language-expressible name, is one clear indicator that we have a mental image, possibly a semantic category, for such a segment. Other indicators might exist but are not needed in the context of the here-asked questions.

There are, however, indeed some segments that cannot be named and still “have a meaning for us.” One example is the concave segment found on many plastic bottles used to close the hand around it when lifting the bottle. Hence, name-ability does not render a necessary condition for being a meaningful object (or object-part) but it is sufficient and provides at least a strong indicator for this. In addition, we observed that name-ability is correlated with the computer-vision based reliability measure for the segments. The more reliable they are the more often one can name them (Figure 4). Also we have observed that unclear cases which mostly are those where subjects though that these segments could potentially by divided further do not much exist (blue curve in Figure 4).

Thus, the here performed segmentation generically renders identifiable object-*parts* (e.g., “head,” “arm,” “handle” of fridge, etc. Hoffman and Richards, 1984). This is not trivial because segmentations based on other low-level visual features (edges, color, etc.) will not achieve this. On the other hand, arguably no purely data-driven method exists, which would allow detecting complex, *compound* objects (e.g., “woman”) as this requires additional conceptual knowledge. Also, one observes that the actual name for an object(part) depends on scene-context and on each subject's background knowledge. These cognitive aspects, which relate to context analysis, hierarchization, categorization, and other complex processes (Logothetis and Sheinberg, 1996), however, are not relevant here; instead it is quite remarkable that a purely geometrical breaking up of a 3D-scene, most often leads to entities for which we have an internal object-part concept which may reflect the low-level perceptual grounding of the “bounded region” hypothesis formulated by Langacker as a possible foundation for grammatical entity construal (Langacker, 1990).

One could try to introduce additional experimental paradigms to address some of the above discussed aspects. Instead of this we refer our readers to the first paper of this series (Wörgötter et al., 2015), where we have addressed the problem of object concepts “as such.” Both studies support each other and suggest that convex-concave transitions play a major role for our understanding of objects and/or object parts.

### 3.1. Conclusion

The central problem with which we are continuously faced is “to make sense” of the multitude of sensory features that arise in a widely varying way even from similar objects. This is especially troubling for young, inexperienced humans, who cannot rely on much prior knowledge. There is increasing, albeit much debated, evidence that core cognitive systems (Spelke et al., 1994) are operational for several complex aspects like “object,” “agent,” “cause,” etc., already in very young infants (see e.g., Carey, 2011 and commentaries therein for a discussion of the nativist vs. empiricist stance on this). This notwithstanding it remains a formidable problem to find a way to bind different sensory features together to allow reliable object segregation. Color, texture and other such statistical image features vary widely (Geisler, 2008); deterministic features (e.g., coherent motion) may be less variable, but normally we do not need them to individuate (and recognize) objects, for example when analyzing a static scene. Hence, none of these features can take a leading role in this process. By contrast, the current set of two papers supports that convex-concave transitions between 3D-surfaces could indeed provide a strong prior to which a contiguous concept of object-ness can be unequivocally bound. This feature reaches across perception and action (see Wörgötter et al., 2015) into our cognitive understanding of objects and their parts (this study), and may help tying to it other less stable sensory aspects of objects.

## Funding

The research leading to these results has received funding from the European Community's Seventh Framework Programme FP7/2007-2013 (Specific Programme Cooperation, Theme 3, Information and Communication Technologies) under grant agreement no. 270273, Xperience.

### Conflict of interest statement

The authors declare that the research was conducted in the absence of any commercial or financial relationships that could be construed as a potential conflict of interest.

## Footnotes

^1^Under special circumstances we certainly can do this and divide a part further. For example a joiner may need to tell her apprentice that the “head” of the chair leg needs polishing and there may even a specific name for the “head.” Laypersons will not even know it and, thus, in everyday speech such subdivisions would hardly ever be made.

## References

[B1] Arbelaez P. Hariharan B. Gu C. Gupta S. Bourdev L. Malik J. (2012). Semantic segmentation using regions and parts, in CVPR (Providence, RI), 3378–3385.

[B2] Arbeláez P. Maire M. Fowlkes C. Malik J. (2011). Contour detection and hierarchical image segmentation. IEEE Trans. Pattern Anal. Mach. Intell. 33, 898–916. 10.1109/TPAMI.2010.161 20733228

[B3] Ben Salah M. Mitiche A. Ayed I. B. (2011). Multiregion image segmentation by parametric kernel graph cuts. IEEE Trans. Image Proc. 20, 545–557. 10.1109/TIP.2010.2066982 20716502

[B4] Bertamini M. Wagemans J. (2013). Processing convexity and concavity along a 2-D contour: figure-ground, structural shape, and attention. Psychon. Bull. Rev. 20, 191–207. 10.3758/s13423-012-0347-2 23188740

[B5] Biederman I. (1987). Recognition-by-components: a theory of human image understanding. Psychol. Rev. 94, 115–147. 10.1037/0033-295X.94.2.115 3575582

[B6] Boykov Y. Funka-Lea G. (2006). Graph cuts and efficient n-d image segmentation. Int. J. Comput. Vis. 70, 109–131. 10.1007/s11263-006-7934-5

[B7] Braunstein M. L. Hoffman D. Saidpour A. (1989). Parts of visual objects: an experimental test of the minima rule. Perception 18, 817–826. 10.1068/p180817 2628932

[B8] Carey S. (2011). Précis of ‘the origin of concepts’ (and commentaries). Behav. Brain Sci. 34, 113–167. 10.1017/S0140525X10000919 21676291 PMC3489495

[B9] Cate A. D. Behrmann M. (2010). Perceiving parts and shapes from concave surfaces. Atten. Percept. Psychophys. 72, 153–167. 10.3758/72.1.153 20045886 PMC2805109

[B10] Comaniciu D. Meer P. (2002). Mean shift: a robust approach toward feature space analysis. IEEE Trans. Pattern Anal. Mach. Intell. 24, 603–619. 10.1109/34.1000236

[B11] Felzenszwalb P. Huttenlocher D. (2004). Efficient graph-based image segmentation. Int. J. Comput. Vis. 59, 167–181. 10.1023/B:VISI.0000022288.19776.77

[B12] Geisler W. (2008). Visual perception and the statistical properties of natural scenes. Annu. Rev. Psychol. 59, 167–192. 10.1146/annurev.psych.58.110405.085632 17705683

[B13] Gupta S. Arbelaez P. Malik J. (2013). Perceptual organization and recognition of indoor scenes from RGB-D images, in CVPR (Portland, OR), 564–571.

[B14] Hoffman D. Richards W. (1984). Parts of recognition. Cognition 18, 65–96. 10.1016/0010-0277(84)90022-2 6543164

[B15] Jeannerod M. (1994). Development of reaching and grasping, in Motor Development in Children, eds Fedrizzi E. Avanzini G. Crenna P. (John Libbey & Company), 25–32.

[B16] Koenderink J. J. van Doorn A. J. (1982). The shape of smooth objects and the way contours end. Perception 11, 129–137. 10.1068/p110129 7155766

[B17] Langacker R. W. (1990). Concept, Image, and Symbol: The Cognitive Basis of Grammar. Berlin; New York, NY: Mouton de Gruyter.

[B18] Logothetis N. K. Sheinberg D. L. (1996). Visual object recognition. Annu. Rev. Neurosci. 19, 577–621. 10.1146/annurev.ne.19.030196.003045 8833455

[B19] Mandler J. M. (1992). How to build a baby: II. Conceptual primitives. Psychol. Rev. 99, 587–604. 10.1037/0033-295X.99.4.587 1454900

[B20] Mandler J. M. (2012). On the spatial foundations of the conceptual system and its enrichment. Cogn. Sci. 36, 421–451. 10.1111/j.1551-6709.2012.01241.x 22435402

[B21] Moosmann F. Pink O. Stiller C. (2009). Segmentation of 3D lidar data in non-flat urban environments using a local convexity criterion, in Intelligent Vehicles Symposium (Xi'an), 215–220.

[B22] Palmeri T. J. Gauthier I. (2004). Visual object understanding. Nat. Rev. Neurosci. 5, 291–303. 10.1038/nrn1364 15034554

[B23] Papon J. Schoeler M. Wörgötter F. (2013). Voxel cloud connectivity segmentation - supervoxels for point clouds, in Proceedings IEEE Conference Computer Vision and Pattern Recognition (CVPR) (Portland, OR), 2027–2034.

[B24] Richtsfeld A. Morwald T. Prankl J. Zillich M. Vincze M. (2012). Segmentation of unknown objects in indoor environments, in Proceedings IEEE Conference EEE/RSJ International Conference on Intelligent Robots and Systems (IROS) (Vilamoura), 4791–4796.

[B25] Riesenhuber M. Poggio T. (2000). Models of object recognition. Nat. Neurosci. 3(Suppl.), 1199–1204. 10.1038/81479 11127838

[B26] Rosin P. L. (2000). Shape partitioning by convexity. IEEE Trans. SMC Part A Syst. Hum. 30, 202–210. 10.1109/3468.833102

[B27] Rubin E. (1958). Visuell wahrgenommene figuren. Copenhagen: Gyldenalske Boghandel 1915, in Readings in Perception, eds Beardslee D. C. Wertheimer M. (Princeton, NJ: Van Nostrand), 194–203. Reprinted as: Figure and Ground.

[B28] Schoeler M. Papon J. Wörgötter F. (2015). Constrained planar cuts - object partitioning for point clouds, in Proceedings IEEE Conference Computer Vision and Pattern Recognition (CVPR) (Boston, MA).

[B29] Siddiqi K. Kimia B. B. (1995). Parts of visual form: computational aspects. IEEE Trans. Pattern Anal. Mach. Intel. 17, 239–251. 10.1109/34.368189

[B30] Silberman N. Hoiem D. Kohli P. Fergus R. (2012). Indoor segmentation and support inference from RGB-D images, in Proceedings European Conference an Computer Vision (ECCV) (Firenze), 746–760.

[B31] Smisek J. Jancosek M. Pajdla T. (2011). 3D with kinect, in Proceedings International Conference on Computer Vision (ICCV) (Barcelona), 1154–1160.

[B32] Spelke E. S. Katz G. Purcell S. E. Ehrlich S. M. Breinlinger K. (1994). Early knowledge of object motion: continuity and inertia. Cognition 51, 131–176. 10.1016/0010-0277(94)90013-2 8168357

[B33] Stein S. Papon J. Schoeler M. Wörgötter F. (2014a). Object partitioning using local convexity, in Proceedings IEEE Conference Computer Vision and Pattern Recognition (CVPR) (Columbus, OH), 304–311.

[B34] Stein S. Wörgötter F. Schoeler M. Papon J. Kulvicius T. (2014b). Convexity based object partitioning for robot applications, in IEEE International Conference on Robotics and Automation (ICRA) (Hong Kong), 3213–3220.

[B35] Ückermann A. Haschke R. Ritter H. (2012). Real-time 3D segmentation of cluttered scenes for robot grasping, in Proceedings IEEE Conference on Humanoids (Osaka).

[B36] Vaina L. M. Zlateva S. D. (1990). The largest convex patches: a boundary-based method for obtaining object parts. Biol. Cybern. 62, 225–236. 10.1007/BF00198097 2302431

[B37] Wörgötter F. Sutterlütti R. Tamosiunaite M. (2015). Perceptual influence of elementary three-dimensional geometry: 1) Object-ness. Front. Psychol. 6:1317 10.3389/fpsyg.2015.01317 PMC4551822 26379613

